# Electro-clinical and neurodevelopmental outcome in six children with early diagnosis of tuberous sclerosis complex and role of the genetic background

**DOI:** 10.1186/s13052-020-0801-0

**Published:** 2020-03-27

**Authors:** M. N. Savini, A. Mingarelli, A. Peron, F. La Briola, F. Cervi, R. M. Alfano, M. P. Canevini, A. Vignoli

**Affiliations:** 1 Child Neuropsychiatric Unit - Epilepsy Center, San Paolo Hospital, Department of Health Sciences, Università degli Studi di Milano, Via di Rudinì 8, 20142 Milan, Italy; 2 Child Neuropsychiatric Unit - Epilepsy Center, San Paolo Hospital, Milan, Italy; 3 Department of Pediatrics, Division of Medical Genetics, University of Utah School of Medicine, Salt Lake City, UT USA; 4 Human Pathology and Molecular Pathology Unit, San Paolo Hospital, Milan, Italy

**Keywords:** Tuberous sclerosis complex, TSC1, TSC2, Seizure, Infancy, Electroencephalography, Neurodevelopmental outcome

## Abstract

**Background:**

Seizures in individuals affected by tuberous sclerosis complex (TSC) commonly develop in the first year of life, are often preceded by a progressive deterioration of the electroencephalogram (EEG), and likely influence developmental outcome. Although early diagnosis of TSC has offered a tremendous opportunity to monitor affected patients before seizure onset, reports of the neurological manifestations of TSC in infants before seizure onset are still scarce. Here we describe early EEG activity, clinical and genetic data and developmental assessment in a group of TSC infants, with the aim of identifying possible prognostic factors for neurodevelopmental outcome.

**Methods:**

We report on six infants diagnosed with TSC pre- or perinatally, who underwent serial Video-EEG recordings during the first two years of life. EEGs were classified based on distribution and intensity of interictal epileptiform discharges, and Vigabatrin was introduced in case of ictal discharges. Psychomotor development, cognitive functioning and behavioral problems were assessed through standardized scales. Molecular testing included analysis for point mutations and deletions/duplications in *TSC1* and *TSC2*.

**Results:**

EEG abnormalities appeared at a mean age of 4 months. Four of the six patients developed seizures. EEG abnormalities preceded the onset of clinical seizures in all of them. The two individuals with good seizure control showed normal development, while the other two exhibited psychomotor delays. The patients who did not develop seizures had normal development. A pathogenic variant in the *TSC2* gene was detected in all patients but one. The one without a mutation identified did not develop seizures and showed normal neurodevelopment. Of note, the two patients presenting with the worst outcome (that is, poor seizure control and intellectual/behavioral disability) both carried pathogenic variants in the GAP domain of *TSC2*.

**Conclusion:**

Our report supports the importance of EEG monitoring before seizure onset in patients with TSC, and the correlation between prompt seizure control and positive neurodevelopmental outcome, regardless of seizure type. Our results also indicate a possible role of the genetic background in influencing the outcome.

## Introduction

Tuberous Sclerosis Complex (TSC) is a multisystem neurocutaneous disorder caused by heterozygous pathogenic variants in *TSC1* (Chr. 9q34.13) or *TSC2* (Chr. 16p13.3) [1]. It is characterized by hamartomas affecting the brain, skin, eye, heart, lung, and kidney. The typical Central Nervous System (CNS) lesions are present in more than 90% of the patients and consist of cortical tubers, white matter radial migration lines, subependymal nodules (SENs), and subependymal giant cell astrocytomas (SEGAs) [1]. Up to 85% of the affected individuals have a diagnosis of epilepsy, and seizure onset in the first year of life is very common (67%) [2]. TSC may therefore be considered a genetic developmental and epileptic encephalopathy [3], as both genetic factors and epileptic activity contribute to the phenotype. Early onset epilepsy usually presents with focal seizures or infantile spasms, but all seizure types have been clinically described [4, 5].

There is a strong association between intellectual disability (ID) and epilepsy [6], and it is recognized that early seizure onset may negatively contribute to worsening the final developmental outcome. On the other hand, the influence of subclinical seizures has yet to be clarified [3, 5].

Recent studies have shown that many infants develop a progressive deterioration of the EEG during the first months of life, before the onset of seizures [3, 7, 8]. Furthermore, clinically silent seizures can be present in patients with TSC, and are difficult to detect unless an ictal EEG is available [9, 10].

Close EEG monitoring (i.e. every 4–6 weeks) during the first months of life allows for early detection of electroencephalographic seizures and for prompt treatment to minimize the deleterious impact of early-onset seizures [11, 12].

Nowadays, a diagnosis of TSC may be suspected prenatally if cardiac rhabdomyomas are detected on fetal ultrasound, and is subsequently confirmed soon after birth by the presence of skin findings [13], brain findings, or molecular testing. Thus, early diagnosis has allowed clinicians to follow children with TSC before the development of neurological signs. To the best of our knowledge, there are only few reports of the neurological manifestations of TSC in infants before seizure onset. In this study, we describe early EEG activity, clinical and genetic data, and developmental outcome in order to identify prognostic factors that could lead to a better management of these children.

## Material and methods

We performed a retrospective study of children referred to our TSC clinic, which now comprises over 200 individuals. We selected those who had been suspected to have TSC prenatally, perinatally, or in the first 6 months of life and had been followed at our clinic on a regular basis since 2013. A definite diagnosis of TSC was established after birth according to the 2012 revised criteria [1].

Our standard clinical practice consists of serial Video-EEG recordings every 4–8 weeks during the first two years of life. In addition to video-EEG monitoring, parents are educated on how to recognize and video record subtle seizures and spasms, in case seizures develop in between two recordings.

If seizures occur, antiepileptic treatment with Vigabatrin is introduced, starting from 50 mg/Kg/day up to 100–150 mg/Kg/day, according to the current clinical recommendations [14]. Patients with normal EEG recordings are followed up without any antiepileptic medications.

Scalp-EEGs with synchronized video are recorded according to the International 10–20 system, modified for newborns using our Institution’s clinical EEG software (Micromed, System plus). All EEG recordings include at least the following channels (Fp2-C4, C4-O2, Fp2-T4, T4-O2, Fp1-C3, C3-O1, Fp1-T3, T3-O1, T4-C4, C4-Cz, Cz-C3, C3-T3), along with electrocardiography (ECG), deltoid muscles, and breathing recordings. In infants, EEGs are recorded applying the International 10–20 system of electrode placement directly or by cable telemetry with more than 16 channels and at least 3 polygraphic channels (ECG and deltoid muscles).

Prolonged recordings (at least 90 min) during wakefulness and sleep were obtained for all patients.

We reviewed and analyzed each EEG for:

- Background activity appropriate for age, and normal sleep pattern (i.e. presence of sleep spindles, tracè alternante for the newborn);
- Presence of epileptiform discharges (focal, multifocal, generalized);
- Presence of hypsarrhythmia;
- Presence of electroclinical seizures.

We analyzed EEG abnormalities applying the classification proposed by Domanska-Pakiela et al. [7], which is based on interictal epileptiform discharges’ (IEA) distribution and their intensity. EEGs were analyzed by two epileptologists (AM and AV), who were blinded to the patients’ management. Details of the methods used for analyzing EEGs are presented in Table 1. All the patients underwent brain MRI studies performed with a 1.5 Tesla magnet, including fluid-attenuated inversion recovery (FLAIR) sequences to detect the presence of typical cerebral lesions (cortical tubers, subependymal nodules, white matter migration lines, and SEGA).

**Table 1:** EEG’s abnormalities classification

Distribution of IEA	Intensity of IEA
A One region (one focus)	1 Irregular spikes or sharp waves
B One hemisphere (> 1 focus)	2 Irregular spike and wave complexes
C Multifocal (at least 2 foci, each in a different hemisphere)	3 Continuous spike and waves complexes
D Generalized	4 Hypsarrhythmia

Psychomotor development (total Developmental Quotient: DQ) or cognitive level (total Intelligence Quotient: IQ) was assessed through standardized scales (Griffiths’ Scales of Infant Development, GMDS-ER, Wechsler Preschool and Primary Scales of Intelligence, WPPSI). Behavioral problems were investigated through prolonged clinical observations, caregivers’ reports, and standardized tests in children.

Molecular genetic testing for *TSC1/TSC2* was available for all the patients, and included analysis for point mutations and deletions/duplications in both genes. All variants identified were submitted to a publicly available database, whenever not already present (http://chromium.lovd.nl/LOVD2/TSC/home.php).

## Results

The study cohort consisted of 6 children (4 boys and 2 girls). The diagnostic criteria that were present in each patient at time of enrolment are reported in Table 2.

**Table 2:** Patients’ characteristics

Patient	Gender	TSC Criteria at enrollment	Other MRI findings	Genetic testing	Age at first EEG	Result	Age at abn EEG (A)	Classification	Age at Sz onset (B)	Sz type	Time from A to B	GVG	Sz Relapse	EEG at f-up	EEG abn-N	Age at last f-up	Cognitive-behavioral phenotype
1	F	PsCT,CT,SENs		*TSC2*	4 m	N	8 m	c1	na	na	na	na		N		25 m	N
2	M	PsCT,HM,CT,SENs		NMI	6 m	N	na	N	10 m	CS (fever)	na	na		N		36 m	N
3	M	PCT,CT,SENs,RH		*TSC2*	3 m	N	4 m	c3	4 m	IS	0	4 m		N at 6 m	2 m	25 m	mild LD
4	F	PCT,CT,SENs		*TSC2*	2 m	N	4 m	d3	7 m	IS	3 m	7 m		N at 12 m	5 m	30 m	N
5	M	PsCT,CT,SENs	SEGA	*TSC2*	1 m	FD	1 m	d3	4 m	IS, (FS 8 m)	3 m	4 m	20 m	FD		52 m	ID; ASD
6	M	PCT,CT	MCD	*TSC2*	2 m	N	4 m	b2	4 m	FS, (IS 7 m)	0	4 m	5 m	FD		25 m	ID

*MRI* magnetic resonance imaging, *SZ* seizure, *PCT* Prenatally found cardiac tumors, *PsCT* Postnatally found cardiac tumors, *HM* hypomelanotic macules, *RH* retinal hamartomas, *CT* cortical tubers, *SENs* subependymal nodules, *MCD* malformation of cortical development, *NMI* No Mutation Identified, *N* normal, *na* not applicable, *abn* abnormal, *FD* focal discharges, *CS* clonic seizure, *FS* focal seizure, *IS* infantile spasms, *GVG* Vigabatrin, *LD* language delay, *ASD* autism spectrum disorders.

Time from A to B: Time from age at abnormal EEG to age at seizure onset

The diagnosis was suspected following the detection of cardiac rhabdomyomas (3 prenatally, 2 perinatally, and 1 at age 6 months) in all the patients. Subsequent skin examination, neuroimaging, eye examination and abdominal ultrasonography showed the presence of hypomelanotic macules, retinal hamartomas, cortical dysplasia (including tubers and cerebral white matter radial migration lines) and subependymal nodules as the second major diagnostic criterion. No renal angiomyolipomas have been detected in these young patients yet, whereas in three individuals renal cysts were present at the time of evaluation (Patients n. 3, 5 and 6).

Genetic testing documented pathogenic variants in *TSC2* in all but one patient. In one individual no mutations in *TSC1/TSC2* were identified.

Age at first evaluation ranged from 4 weeks to 6 months, depending on different referrals, with a follow-up of 2–4 years.

### Patient 1

Patient 1 was born at 37 gestational weeks by caesarean section for breech presentation, after an uneventful pregnancy. Multiple cardiac rhabdomyomas were detected in the neonatal period. Cardiac arrhythmias were recorded on 24-h EKG Holter monitoring, and treatment with Beta-blockers and Flecainide acetate was started.

**Fig. 1:**
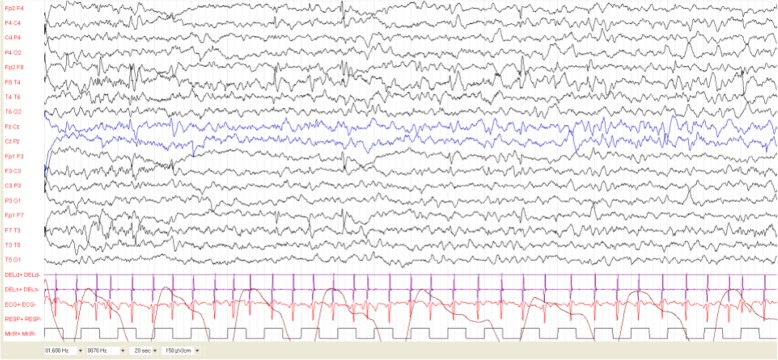
Patient 1 at age 12 months. EEG recording shows normal background activity with multifocal spikes and sharp waves during sleep, involving the fronto-temporal regions of both hemispheres. The child did not experienced seizures. Interictal discharges disappeared by age 24 months

The first EEG was performed at age 4 months and was normal. EEGs performed every 8 weeks showed the presence of normal background activity with multifocal spikes and sharp waves in sleep, involving the occipital left region (since age 8 months), the right fronto-temporal region (since age 12 months), or the left fronto-temporal region (18 months). We classified Patient 1 as C1 [Fig. 1]. Interictal discharges disappeared by age 24 months. She never experienced epileptic seizures.

Neurological examination and psychomotor development were normal at two years of age (DQ 98, Griffiths Scales). Genetic testing showed a pathogenic variant in exon 24 of *TSC2*: c.2771_2772del (p.Phe924*).

### Patient 2

Patient 2 was born at 32 weeks by caesarean section, after a pregnancy complicated by maternal hypertension and diabetes mellitus type 1. Apgar score was 5–9 and weight was 2360 g. Delivery was complicated by perinatal respiratory distress and pneumothorax requiring continuous positive airways pressure (CPAP) ventilation.

Multiple cardiac tumors were detected at 6 months of age.

The first EEG was performed at age 6 months, and was normal. Follow up EEGs were done every 8 weeks, and remained normal until age 2 years.

At age 10 months he presented with an isolated seizure associated with fever. The seizure was characterized by staring and clonic jerks involving the upper limbs, followed by spontaneous recovery in about 2 min.

Neurological examination and neuropsychological development at 34 months of age were normal (DQ 91, Griffiths Scales).

Molecular analysis for *TSC1* and *TSC2* point mutations (through next generation sequencing) and deletions/duplications was negative.

### Patient 3

Patient 3 was born at 37 weeks of gestation by vaginal delivery. Multiple cardiac tumors had been detected on prenatal ultrasonography at 32 weeks of gestation, and were confirmed by fetal MRI. The first EEGs were performed during the neonatal period and at 3 months of age at a different institution, and were normal.

Seizures occurred at age 4 months and the patient was therefore referred to our TSC clinic. They were characterized by series of infantile spasms, with focal motor signs and asymmetric involvement of the upper limbs (more on the left side) documented on parents’ video recordings. EEG was performed, and showed interictal discharges characterized by frequent multifocal spikes and spike-and-waves complexes during wakefulness and sleep involving mainly the right centro-temporal region. We did not record hypsarrhythmia. We classified this patient’s EEG features as C3. The patient was started on Vigabatrin at 75 mg/Kg/day, and seizures have been controlled since the first day of treatment. He has been seizure free to the time of last assessment.

Follow-up EEG performed at age 6 months (2 months after starting Vigabatrin) was normal, and remained normal through age 17–24 months.

At two years of age, neurological examination and neuropsychological assessment were normal (DQ 89, Griffiths Scales), with mild language delay.

Genetic tests identified a large deletion involving exons 17–22 of *TSC2*.

### Patient 4

Patient 4 was born at 40 weeks by vaginal delivery after an uneventful pregnancy. Multiple cardiac rhabdomyomas had been detected prenatally. The first EEG was performed at age 8 weeks and was normal. Follow up EEG performed 8 weeks later showed focal spikes and spike waves complexes during sleep over the right central region. The option of preventive antiepileptic treatment with Vigabatrin was proposed, but the family decided to wait and delayed the appointment for EEG recordings.

Three months later, at age 7 months, the parents reported a series of infantile spasms on awakening.

**Fig. 2:**
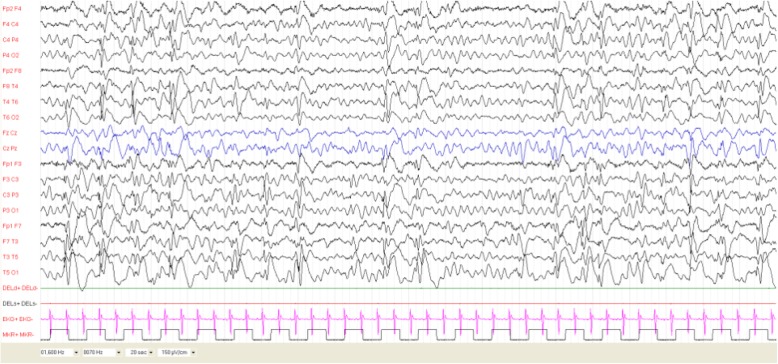
Patient 4 at age 7 months. EEG shows frequent interictal discharges, characterized by diffused spike and wave discharges. At this time, she experienced epileptic spasms and she was treated with Vigabatrin. Seizures were promptly controlled. At follow-up EEGs were completely normal

EEG was performed, and showed frequent interictal discharges, characterized by multifocal spike and wave discharges with diffusion during sleep. We never recorded the presence of hypsarrhythmia. This patient was classified as D3 [Fig. 2].

She was treated with Vigabatrin (100 mg/Kg/day), with good seizure control since the first week of treatment.

Follow-up EEGs showed progressive decrease of epileptic discharges at age 8, 10 and 12 months, and were completely normal at serial recordings after the first year of age.

Neuropsychological development at age 28 months was normal (DQ 104, Griffiths Scales). Neurological evaluation was normal.

A pathogenic variant was identified in exon 30 of *TSC2*: c.3626 T > C (p.Leu1209Pro), inherited from her affected mother.

### Patient 5

The child was born by vaginal delivery after an uneventful pregnancy. He presented with cardiac arrhythmias during the neonatal period, and subsequent echocardiography showed multiple cardiac rhabdomyomas. The first EEG was performed at age 4 weeks and was abnormal, showing focal interictal discharges over the right fronto-temporal region. The first EEG was performed at our clinic at 4 months of age, and showed an increase in epileptic discharges with frequent multifocal spike-and-waves complexes during sleep. While the parents were deciding whether or not to start treatment with Vigabatrin, the family noticed a series of infantile spasms four days after the first EEG recording. The patient was admitted to the hospital, and seizures were recorded at video EEG monitoring. They were characterized by series of spasms with ictal EEG correlates of pseudo periodic high voltage generalized slow waves. Interictal EEG showed multifocal spike and spike-and-waves discharges, with bilateral activation and diffusion during sleep. This patient was classified as D3. Treatment with Vigabatrin (titrated to 150 mg/Kg/day) was started, with control of seizures from the 10th day of treatment.

At 8 months of age he experienced seizures associated with fever; valproic acid was added, with good seizure control.

At age 20 months the patient started to present polymorphic seizures, characterized by prolonged focal clonic seizures involving the left arm during febrile illnesses, with or without bilateral tonic-clonic evolution. Relapsing epileptic spasms coexisting with focal subtle seizures were documented from age 20 months. He was treated with several AEDs with unsatisfactory results. Epilepsy is still drug-resistant at the time of last assessment.

Follow up EEG performed at age 6, 8 and 12 months showed persistent multifocal discharges.

At age 4 years a SEGA of 13 × 10 mm located near the left foramen of Monro was detected on brain MRI, which was stable at neuroradiological follow-up.

At the time of last assessment, the patient’s psychomotor development was delayed and moderate cognitive impairment was diagnosed (IQ 48, WPPSI at age 4 years). Autism spectrum disorder (ASD) was also diagnosed (ADOS evaluation).

Genetic testing showed a pathogenic variant in exon 34 of *TSC2*: c.4544_4547delACAA (p.Asn1515Serfs*60).

### Patient 6

The patient was born at term after an uneventful pregnancy. Multiple cardiac tumors had been detected prenatally. Brain MRI showed bilateral cortical and cerebellar tubers, and a large malformation of cortical development involving the fronto-temporal bilateral regions.

The first EEG performed at age 8 weeks was normal.

At age 4 months, he started to present focal seizures characterized by staring, head and eye deviation, with sparing of the left arm.

Focal seizures arising from the right frontal region were recorded at Video-EEG. Interictal EEG was characterized by focal spike and spike-and-waves discharges on the right fronto-temporal region.

At 7 months of age the patient started to present infantile spasms associated with subtle focal seizures. Sleep EEG was characterized by diffuse slow activity with sporadic spike and wave discharges predominantly on the right fronto-temporal region, without hypsarrhythmia.

**Fig. 3:**
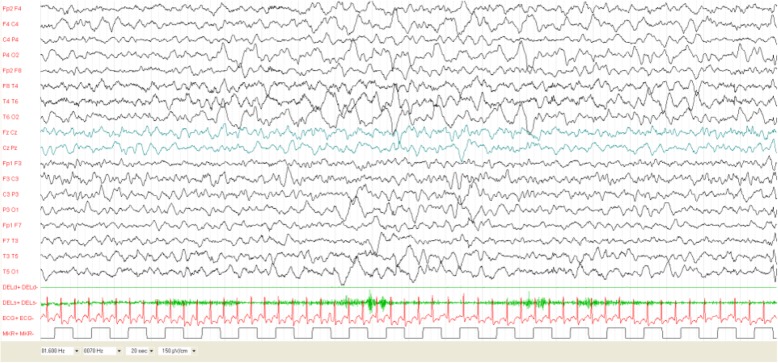
Patient 6 at age 7 months. Sleep EEG was characterized by diffuse slow activity with sporadic spike and wave discharges predominantly on the right fronto-temporal region. He showed both focal seizures and epileptic spasms

The patient was classified as B2 [Fig. 3].

Treatment with Vigabatrin initially introduced for focal seizures, was titrated up to 120 mg/Kg/day. Due to drug-resistant seizures, Levetiracetam, Topiramate and Carbamazepine in different combinations were introduced with unsatisfactory results.

Follow-up EEGs confirmed the presence of persisting interictal discharges that were predominant over the right fronto-temporal regions.

Patient 6’s psychomotor development was delayed based on neuropsychological testing performed at age 24 months (DQ 69, Griffiths Scales), with predominant speech delay.

Genetic testing showed a de novo pathogenic variant in exon 35 of *TSC2:* c.4570dup (p.Ser1524Serfs*5).

**Fig. 4:**
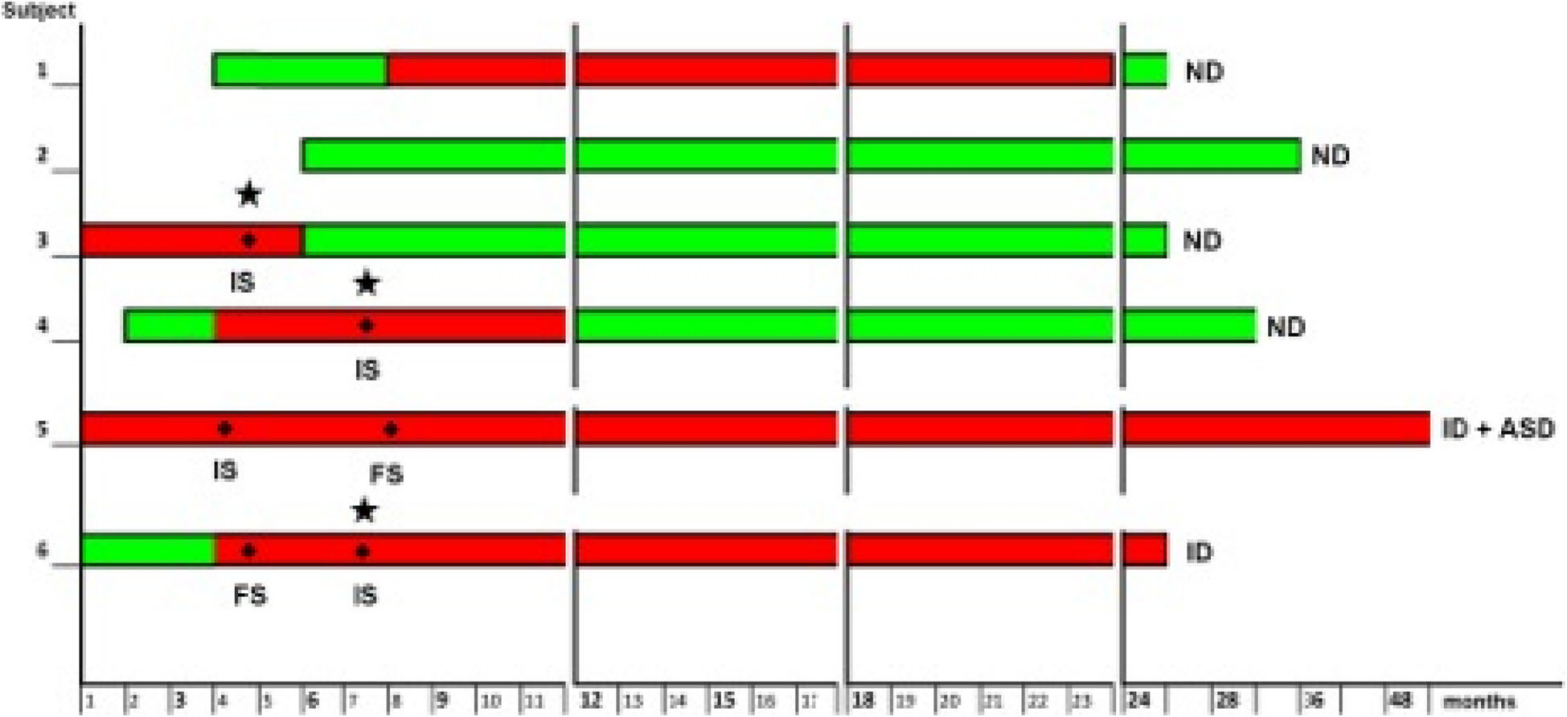
Course of EEG activity and epilepsy for each patient: the green bar represents normal EEG, while the red one the occurrence of EEG abnormalities. ND = Normal Development, ID = Intellectual Disability, ASD = Autism Spectrum Disorder, IS = Infantile Spasms, FS = Focal Seizures. The star indicates drug responsive seizures

The course of EEG activity and epilepsy for each patient is reported in Fig. 4.

## Discussion

Most Authors recognize a link between early seizure onset and poor long-term neurological and behavioural outcome [15]. This has stimulated several investigators to develop strategies to prevent seizure onset [12]. Assuming that interictal epileptiform abnormalities (IEA) in children with TSC can be considered a marker of the dynamics of the epileptogenic process [7], multicenter prospective randomized double-blind clinical trials are currently ongoing, aiming at verifying the impact of the treatment of preclinical vs. clinical seizures (EPISTOP project in Europe and PREVENT trial in the United States [NCT02849457]). However, data about early EEG monitoring in TSC patients are limited in the literature [7, 15].

While waiting for the results of these seminal studies, in clinical practice we are faced with newborns referred for TSC due to the improvement of ultrasound techniques during pregnancy. Nowadays TSC is increasingly more frequently diagnosed in the prenatal period or in early infancy, thus offering the possibility of monitoring children before the onset of seizures and/or neurodevelopmental delay [16].

According to the clinical recommendations for management of epilepsy in TSC [14], treatment should be initiated in infants and children within 24 months of age if ictal discharges occur, with or without clinical manifestations.

Since 2013, we have had the opportunity to follow 6 infants with TSC starting from the first months of life. All of them received video-EEG recordings every 4–8 weeks. The aim of the present study was to describe the electro-clinical and neurodevelopmental outcome in 6 children with early diagnosis of TSC.

Although limited by the small number of individuals included in this study and the relatively short follow-up, neither age at EEG abnormalities appearance nor EEG characteristics at onset were sufficient for determining long-term prognosis. Indeed, one of the patients described herein showed EEG abnormalities for months and never developed seizures (Patient 1). Her neurodevelopment is normal. On the other hand, disappearance of EEG abnormalities was related to good prognosis for both epilepsy and cognitive outcome.

Mean age at EEG abnormalities appearance was 4 months, in line with that reported in larger cohorts by Jozwiack et al. [12].

According to the EEG classification proposed by Domanska-Pakiela et al. [7], irregular and continuous spike-and-waves complexes, either localized on one hemisphere or multifocal, were more frequently seen also in our group of patients. Clear hypsarrhythmia was not identified on the EEGs even though four infants showed IS.

The lack of EEG deterioration could be explained by the close EEG monitoring leading to early treatment of IS, which did not allow the progression of epileptiform activity [7]. This finding should be considered a typical feature in TSC, and clinicians should not wait to start antiepileptic treatment [8].

In addition, seizure type - either focal seizures or IS - did not correlate with epilepsy or intellectual prognosis in our sample.

The major contribution to neurodevelopment seems not only linked to seizure onset but rather to the opportunity of their prompt control, as seen in patients 3 and 4 from our cohort. This finding is in line with the recent work by Capal et al. [17], demonstrating that timing of complete seizure control, in particular at age 12 months, is predictive of global development and ASD behaviors in the long-term.

Moreover, our data further confirm that the refractoriness of seizures is associated with cognitive disability, as seen in Patients 5 and 6 and reported by Humphrey et al. [18], who demonstrated that IQ decline is associated with duration of infantile spasms in infants with TSC.

Nevertheless, it is known that seizures are not the only determinant of cognitive status in TSC. Genetic factors, i.e. pathogenic variants in *TSC2* rather than *TSC1* [19], have been recognized as a significant risk factor for earlier and more severe epilepsy, as well as for a higher rate of cognitive impairment, with due exceptions [20, 21].

Specifically, *TSC2* pathogenic variants have been associated with a significantly higher occurrence of IS and other epilepsy types. However, certain missense mutations located in the central region of *TSC2* (exons 23–33) have been reported to be associated with a reduced incidence of IS [22]. In fact, we are aware of patients with central missense mutations presenting with IS. Of note, Patient 4 in our cohort exhibited a missense variant in exon 30 of *TSC2* and presented with infantile spasms, but had normal development. This suggests that, although infantile spasms can be seen in patients with central missense mutations (yet, significantly less frequently than in patients with different *TSC2* pathogenic variants), developmental outcome could be favorable.

Our small group of children with TSC was genetically homogenous. A pathogenic variant in *TSC2* was detected in all but one: the latter did not develop seizures and has normal neurodevelopment. On the other hand, the two patients with the worst outcome, in terms of seizure control and intellectual/behavioral disability, both carried pathogenic variants in exons 34 and 35 of *TSC2*.

As hypothesized by Kothare and colleagues [23], changes in the *TSC2* GAP domain may lead to a more pronounced mTOR hyperactivation and a more severe phenotype. We therefore hypothesize that pathogenic variants in exons 34–35 of *TSC2* (GAP domain) are responsible for the worst outcome in the two infants in our sample, but this finding should be confirmed in bigger cohorts.

Taken together, these observations led us to infer that genotype itself could influence the outcome in patients with TSC, at times independent from epilepsy onset, seizure type, and seizure control. However, further studies on larger numbers of patients are warranted to verify this hypothesis.

In addition to the influence of seizures and genetics on the outcome, neuropathological findings, such as a higher tuber/brain proportion [6] or the number of radial migration lines [24] have been demonstrated to contribute to cognitive and behavioural phenotype. All our patients showed multiple cortical tubers and subependymal nodules, as well as white matter radial migration lines on brain MRI, so that this finding was difficult to correlate to the patients’ outcome. The fact that intellectual dysfunction has been reported in patients without epilepsy [5] or with no apparent brain lesions [25], as well as in animal models of TSC [26], suggests that the neurological and behavioral problems in TSC may somehow emerge independently from seizure onset, and be presumably related to underlying altered CNS development and neuronal connectivity.

Nevertheless, epileptic activity and brain structural alterations may also interact with each other, and seizures may result as a trigger for predisposed structures or provide additional insult worsening intellectual disability, as recently suggested [17].

Both early onset seizures and CNS abnormalities in TSC are genetically determined through a cascade of events involving early mTOR overactivation.

This consideration may open the way to the possibility of early treatment with mTOR inhibitors for TSC-related neuropsychiatric disorders. However, in experimental models, animals that were treated prenatally with mTOR inhibitors exhibited long-term cognitive sequelae [27]. On the other hand, administration of mTOR inhibitors in the early postnatal period resulted in preventing seizure onset or in making seizures cease if given after epilepsy onset [28].

Only limited human data on early postnatal use of mTOR inhibitors are available, suggesting this treatment is scarcely effective in preventing seizure onset [29].

Vigabatrin is recognized to be the most effective treatment for IS and focal seizures starting in infants with TSC. In addition to inhibiting GABA-transaminase and increasing the synaptic concentration of GABA in the brain, it also plays a role in inhibiting mTOR overactivation, and may have an impact on glutamatergic transmission, which is essential for neurodevelopment [30].

Inflammatory mechanisms involving specific cytokines and chemokines are abnormally activated in mouse models of TSC, and inhibition of these mechanisms was associated with a decrease in seizures and improved survival in these mice [31]. Starting from this proof-of-concept, other modulating factors should be further considered in children with TSC, and anti-inflammatory treatments may represent potential therapy for this genetic epilepsy.

## Conclusion

Our report supports the importance of EEG monitoring before seizure onset in TSC patients, and the correlation between seizure control and positive neurodevelopmental outcome. Although we could not find specific predictors, mainly because of the small number of patients analysed, we hypothesize a role of the genetic background in influencing the outcome. Hopefully, ongoing studies on large populations of children with TSC will delineate the key players of neurodevelopmental outcome in the near future.

## Data Availability

The datasets during and/or analysed during the current study available from the corresponding author on reasonable request.
